# Explorative Frequency Analysis of Leaf Temperature Behavior of Maize (*Zea mays* subsp. *mays*) at Water Deficit

**DOI:** 10.3390/plants8040105

**Published:** 2019-04-18

**Authors:** Friederike Kögler, Dirk Söffker

**Affiliations:** Chair of Dynamics and Control, University of Duisburg-Essen, Lotharstr. 1-21, 47057 Duisburg, Germany; dirk.soeffker@uni-due.de

**Keywords:** oscillation, system behavior, water stress, Hilbert–Huang Transform (HHT), Phase Portrait (PP), Short-Term Fourier Transform (STFT)

## Abstract

In this study, different standard frequency analysis (FA) methods are applied to measured leaf temperature data of maize plants (developmental stages EC13–15). These FA methods are used to identify specific behaviors, regularities, and sudden changes in frequencies/amplitudes of data, e.g., in control engineering. The thorough application of different FA methods in plant studies is novel. The aim of this paper is to analyze features of the measured data and to explore the explanatory power of different methods for the detection of plant dynamic behavioral changes. The basic assumption is an expected relation between plant water stress and resulting changes in leaf temperature oscillations caused by stress-induced changes in stomatal behavior. Therefore, an irrigation experiment (laboratory; controlled environmental conditions) was implemented to compare leaf temperature behavior of stressed and unstressed plants. Leaf temperature time series are processed and the results are compared as functions of time showing the behavioral changes in terms of the different methods applied. The analysis of results is explained; conclusions, which can be made based on different methods, are given. The study confirms the applicability of FA methods and provides new insights into leaf temperature behavioral patterns. Results are discussed regarding the hypothesized incipience of leaf temperature oscillations due to water stress.

## 1. Introduction

Leaf temperature measurements are utilized to monitor the water status of crops [1,2,3,4]. Leaf temperature depends on climate variables (e.g., air temperature and humidity) and on plant physiological variables (e.g., stomatal aperture) [5,6]. Water availability below maximum plant water demand (water deficit) impacts leaf temperature by the consecutive reduction of transpiration due to stomatal closure [7,8]. Transpiration results in reduced leaf temperatures due to evaporative cooling, particularly if environmental conditions tend to rise considerably leaf temperature, e.g., high air temperatures [5,9]. Hence, reduced transpiration leads to an increase in leaf temperature. Perpetuation of transpiration, in spite of a water deficit, is a key process to regulate leaf temperature under water stress and maintain operativeness of tissues [10,11,12].

The concept of leaf temperature measurement for water status determination is related to the water flow processes through and within the plant [1]. Here, the model of water balance related to these flow processes is based on the thermodynamic water potential relations between soil, plant, and atmosphere as well as between the different compartments within the plant [13]. Water potential is a thermodynamic variable used to express water availability of a system or to model water movements between systems [14]. Here, the driving force for water movements are water potential differences between single compartments. Water and water vapor ‘flow’ from areas of high water potential to areas of low water potential. The detailed interaction between the variables determining plant water balance are complex and still subject of scientific studies [15,16,17].

Relevant for the presented study is the known causality between stomatal aperture, transpiration rate, and leaf temperature to indicate potential water stress [17,18]. Stomatal apertures determine the quantity of plant water transpiration (ceteris paribus), and hence the degree of evaporative cooling of the leaf surface [5,19,20]. According to Webb [21], stomatal apertures are influenced by environmental, physiological, and circadian signals. Major plant external signals are light, CO_2_ concentration, air temperature and humidity, wind speed, and the degree of a water deficit. Major plant internal signals are the concentration of phytohormones, particularly abscisic acid (ABA), and endogenous circadian signals [21,22]. Signals triggering the opening of stomata are low CO_2_ concentrations, high air humidity, wind, and high light intensities; whereas signals triggering the closure of stomata are high CO_2_ concentrations, water deficit, ABA, and darkness [23,24,25].

Further, oscillation of stomatal aperture is described in several studies [21,26,27,28,29,30]. Different oscillation frequencies are attributed to the following major factors.

Circadian rhythms: Oscillations with periods of 23 to 26 h are observed, where stomata of well-watered C3- and C4-plants are open in the days (light) and closed in the nights (darkness) [21,22,26]. Hence, oscillation frequency attributed to circadian rhythms is approximately 1/24 h. Plant internal circadian rhythms are assessed to be a regulation mechanism for physiological responses to external disturbances, e.g., water deficit [21,31].

Water deficit: Oscillations are reported for many species, for example with periods of 15 min to 1 h for maize [26,32], 12–18 min for banana [33], 20 min–1 h for tomato [29], and 30 min–1 h for cotton [34]. These stomatal oscillations were in all cases observed indirectly by measuring either CO_2_-uptake [32], leaf turgor pressure and leaf transpiration [33], water loss in weight [29], or stomatal conductance [34]. Further, oscillating water hydraulic conductance in plants was measured [31]. Assembling the observations, the entire water-bearing system in plants is oscillating autonomously under water deficit [30], and oscillation frequency attributed to water deficits vary between 1/h (period of 60 min) and 5/h (period of 12 min). This behavior is interpreted as synchronized hydraulic signal and at the same time a mechanism to prevent the xylem from cavitation [26,28,29,35]. The latter conclusion is supported by the fact that pumps with oscillating pressurization can provide high pressures also at small flow rates, accompanied by reduced tip pressures [36,37]. Water-related stomatal oscillations are detectable for water-stressed plants but, in some cases, also for well-watered plants [32,33,38].

CO_2_ concentration: Oscillations due to variations of CO_2_ concentration in the air are reported with periods of 2.5 to 5 min for maize [26,32,39], and periods of 5 to 10 min in experiments with Arabidopsis [40]. CO_2_-related oscillations are associated with Ca^2+^ oscillations in guard cells: if air CO_2_ concentration is high, Ca^2+^ oscillation is low (period of 10 min) inducing stomatal closing; if air CO_2_ concentration is high, Ca^2+^ oscillation is higher (period of 5 min) inducing stomatal opening [25,40,41].

Beyond that, for other physiological processes oscillating behavior is described, e.g., for ion channel conductance [28] or plasma membrane electrical properties [42]. These are assumed to have influence on stomatal apertures as well [42].

Target of this study is the identification of leaf temperature oscillation features due to a water deficit. Approach is the application of explorative frequency analysis (FA) methods on experimentally collected leaf temperature data of maize under water deficit conditions. The report is structured as follows. In the next section, materials and methods for the experimental data acquisition and selected FA methods are briefly introduced. Subsequently, the measurements and FA results are presented. Finally, results are discussed and starting points for further investigations are given.

## 2. Materials and Methods

### 2.1. Experimental Setup

Water deficit experiments with young maize plants (*Zea mays*, Ronaldinio, KWS) were conducted on single plant scale under laboratory conditions. Environmental conditions of the experiments were adjusted to remain in fixed ranges: Air temperature at 20–23 °C, air humidity at 20–35%, artificial light (Phillips TC-L, 2 × 75 W, 6500 K, appr. 300 µmol/m^−2^/s^−1^) for LD 16:8 h, artificial ventilation (standard building technology), and fertigation with Seramis © (liquid NPK-fertilizer (1.8%, 1.0%, 2.3%) with micronutrients (0.25 ‰)). Solely irrigation time and water quantity were adjusted automatically.

**Figure 1:**
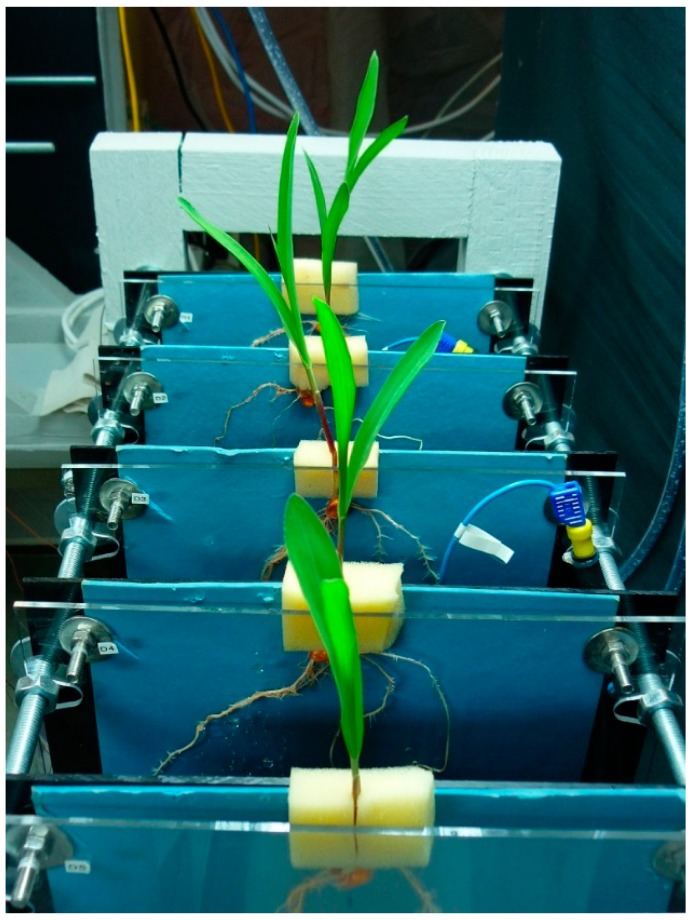
Picture of experimental setup. Maize seedlings grown on individual mounting plates of acrylic glass fixing the plants’ rout system on germination paper. Mounting plates are reversibly fixed in four hangers arranged in front of the infrared (IR) camera.

The presented data result from one experiment with a duration of 10 days. The sample size was 20 individual plants grouped in four groups of five plants each. Before experiment start, plants were grown for nine days in Seramis © substrate. At the three leaf stage (vegetation stage EC 11–13 (US V1–V2)) the seedlings were transferred to individual mounting plates of acrylic glass fixing the plants’ rout system on the germination paper (Hahnemuehlen 3644, 720 g/sqm) (Figure 1). Mounting plates were reversibly fixed in four hangers arranged in front of the Infrared (IR) camera (640 × 480 px, <0.05 °C thermal sensitivity).

Infrared (IR) pictures (sampling period: 20 min) were taken automatically controlled by a programmable logic controller. Leaf temperature values were calculated as follows; original IR camera signals (dimensionless) per pixel and time stamp were extracted from the camera. Measuring points (one pixel in the middle of the second leaf) were selected for all pictures manually from each IR picture. The temperature calculation is based on the original IR camera equations. The results of the first experiment presented below are based on fixed variables for emissivity (0.97 (dimensionless)), air temperature (22 °C), and air humidity (24 %).

### 2.2. Irrigation Regimes

Different irrigation regimes were applied to the four groups: In pretests it was determined that the total water holding capacity of each germination paper sheet amounts to 75–85 g water. Evapotranspiration per day was gravimetrically measured and accounts for approximately 20–25 g/24 h at the beginning of the experiment, increasing to up to 28–33 g/24 h as plants grow. The first days after sowing all plants were fully irrigated until vegetation stage EC 11–13 (US V1– V2) was reached. This point of time is denoted as starting time of the experiment (day 0). The irrigation regime was adjusted for each group: First control group (A) was fully irrigated for the complete period (germination paper bottom in contact with water). Second control group (D) was not irrigated for the complete period. First test group (B) received no water till day 2.5. At this time, the first wilting symptoms were visible (hanging leaf tips and reduced turgor). This state was denoted as mild stress. Irrigation for group B comprised 65 g water per plant (complete refill of total water holding capacity of the germination paper) and was applied manually at day 2.5. Second test group (C) received no water till day 3.5. At this time, serious wilting symptoms were visible (leaves are bend down) and nearly no water (<10 g) was stored in the germination paper. This state was denoted as high stress. Irrigation comprised 80 g water per plant (complete refill of total water holding capacity of the germination paper) as well applied manually at day 3.5. This experimental procedure results in the following basic sets of system states.

- State full irrigation (FI): Amount of available water in the germination paper is 50–85 g. This applies to all plants at the first 1.5 days of the experiment (20 plants) as well as to group A (5 plants) for the total duration of the experiment. Sample size (plants) in state FI: 25.
- State mild stress (MS): Amount of available water in the germination paper is 20–50 g. This applies to groups B, C, and D (15 plants) between day 1.5 and 2.5 of the experiment. Sample size (plants) in state MS: 15.
- State high stress (HS): Amount of available water in the germination paper is 0–20 g. This applies to groups C and D (10 plants) between day 2.5 and 3.5 of the experiment as well as to group D (5 plants) between day 3.5 and the end of the experiment. Sample size (plants) in state HS: 15.

### 2.3. Frequency Analysis Methods

These methods can be used to detect, distinguish, and quantify superimposed or hidden oscillations. By standardized application of these methods to measurement data the specific behaviors, regularities, and sudden changes in frequencies and amplitudes can be analyzed. Further, signal noise, e.g., due to disturbances of measurement devices, can be identified more easily.

Frequency analysis denotes the processing of measurement data to depict the data in another format with specific consideration of frequency and amplitude of oscillations. The graphical representation of these methods simplifies the detection of disturbances and regularities, particularly if the graphs of the test systems are compared to graphs of undisturbed systems. Target of the presented frequency analysis is not the quantification and statistical validation of effects, but rather the qualitative identification of disturbance initiated behavioral changes (i.e., oscillation changes due to water deficits).

Restrictively, according to the Nyquist–Shannon Theorem, the reconstruction of a signal based on equally spaced samples is possible, if the sampling rate equals or exceeds the double of the upper cut-off frequency. This means that frequency properties can only be reliably detected for frequencies fulfilling this theorem. In this study, the sampling period for IR pictures was 20 min (sampling rate: 72/24 h or 1/1200 Hz). Thus, the upper cut-off frequency is certainly less than 36/24 h (1/600 Hz), representing a sampling period of 40 min. Therefore, only the measured frequencies below approximately 36/24h are considered in the following observations.

**Figure 2:**
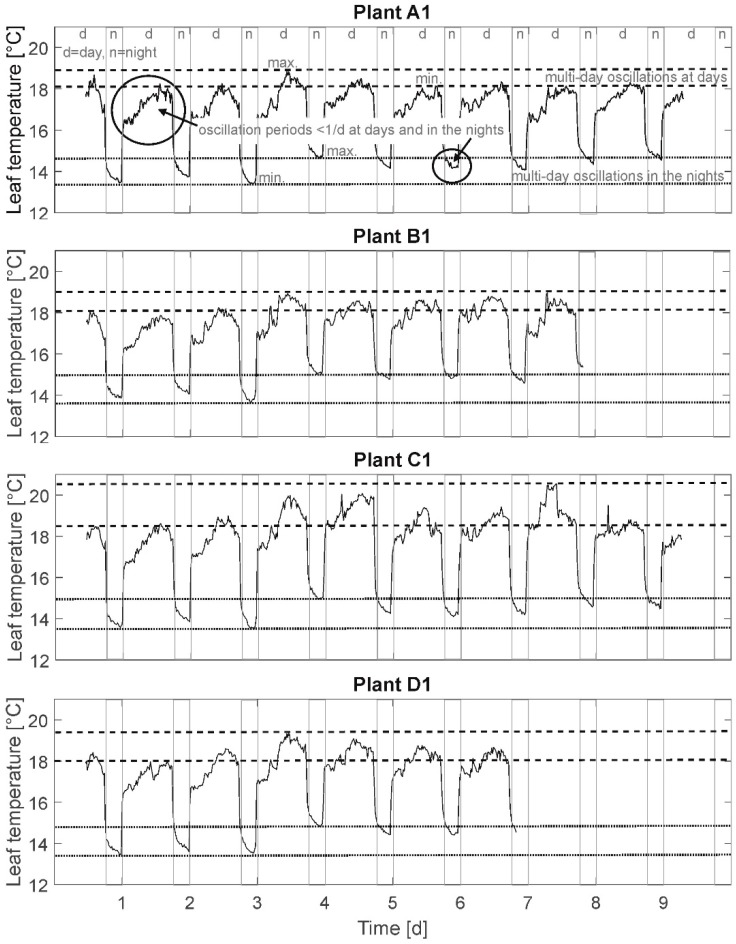
Leaf temperature time series of plants A1, B1, C1, and D1. ││ abrupt temperature changes at illumination switch-on/-off (day–night cycle). - - - oscillations of 1–2 °C amplitudes and 3–4 days periods in the days. ….. oscillations with amplitudes of approximately 1 °C and periods up to 5 days at nights. O oscillations of <1 °C amplitudes and <1d periods in the days and partially also at nights.

Following graphic accounts and explorative frequency analysis methods were applied to the presented leaf temperature time behavior (cf. Figure 2) of the plants:

#### 2.3.1. Local Minima and Maxima (MM)

**Figure 3:**
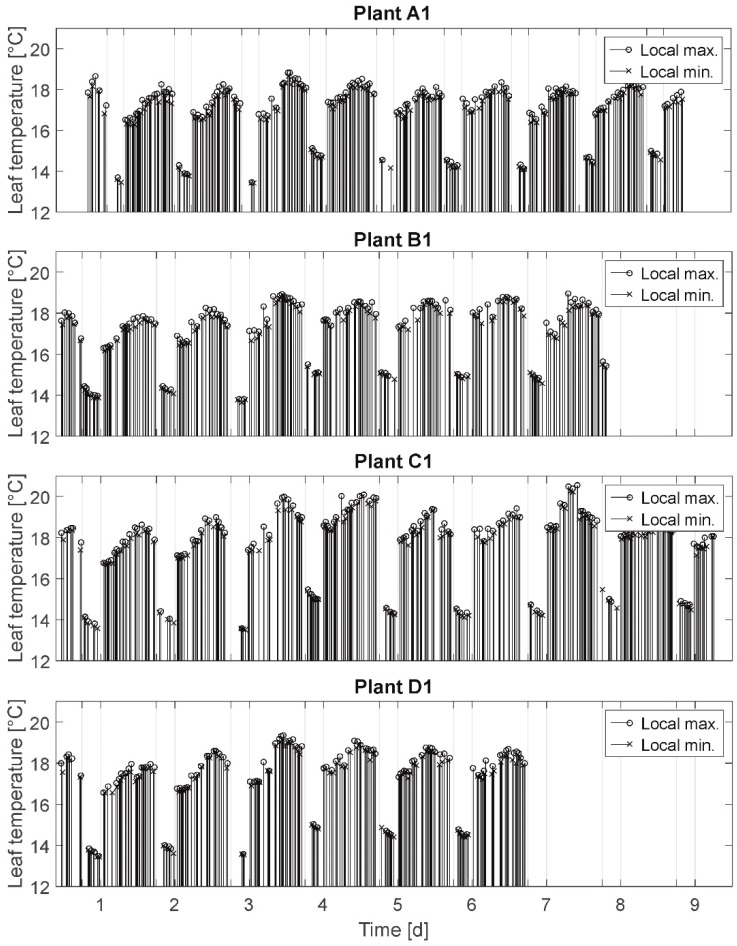
Local minima and maxima of plants A1, B1, C1, and D1. Lines between the x-axis and the signal values denote the events of stomatal opening/closing. Local maxima are attributed to stomatal opening; local minima are attributed to stomatal closure.

Here, the inflection points, calculated by comparison with respectively neighboring raw temperature values, are simply depicted in a diagram to examine by visual comparison of line densities and lengths the differences in oscillation intensities/rhythms (cf. Figure 3). This provides a first qualitative indication of irregularities and differences between the time series which could be investigated further.

#### 2.3.2. Phase Portrait (PP)

**Figure 4:**
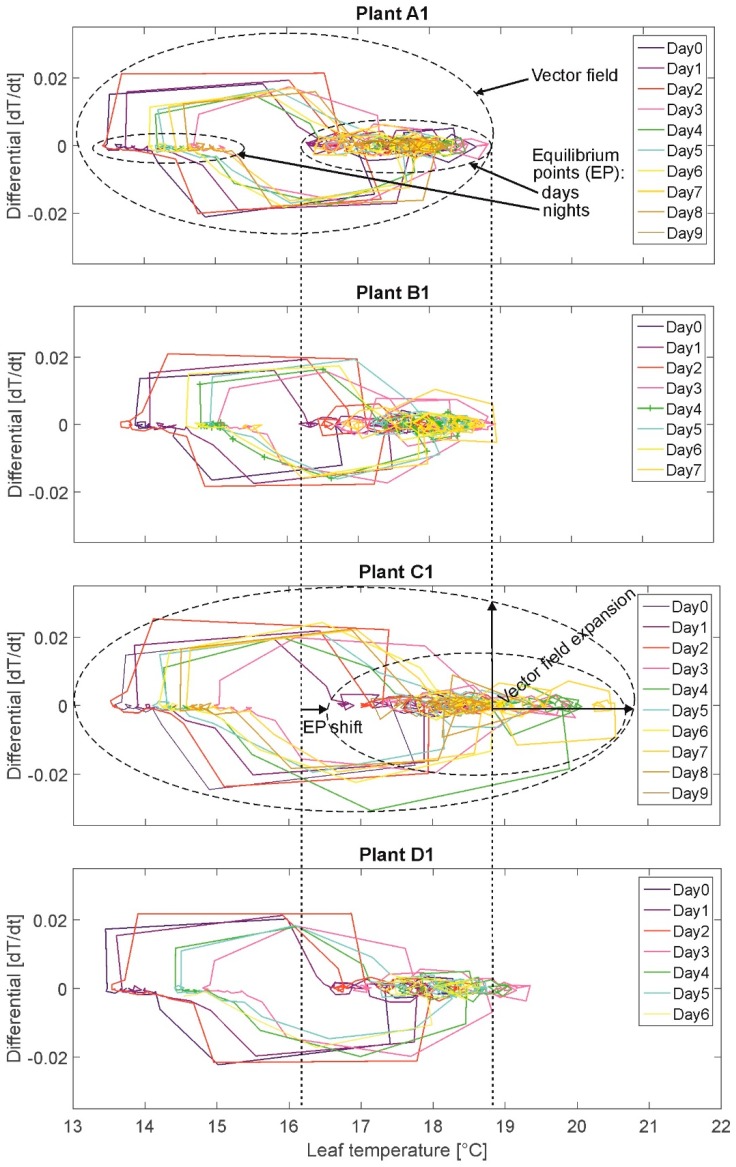
Phase Portrait of plants A1, B1, C1, and D1. Different colors denote different daily cycles. Plotted measurements depict the oscillations by moving in a circle turning clockwise. Small dashed ovals denote equilibrium points (areas) (EP), large dashed ovals denote vector fields.

This method is not a FA method in a closer sense. The PP depicts graphically dynamic system behavior by trajectories (path of changes) of a state variable (here: leaf temperature) in the phase space (here: totality of all measured leaf temperature values). The considered signal value (leaf temperature) is plotted in a coordinate system against its first derivative (leaf temperature change rate, here with Δt = 20 min representing the sampling period) (cf. Figure 4). The comparison of PPs (e.g., of a stressed plant with one of a nonstressed plant) can be used to visualize differences in dynamic system behavior related to equilibrium points (“similar” values occurring often) and vector field (all occurring values). This provides a first visual insight into dynamic properties like periodic time behavior or frequently occurring values.

#### 2.3.3. Cepstrum (CEP)

**Figure 5:**
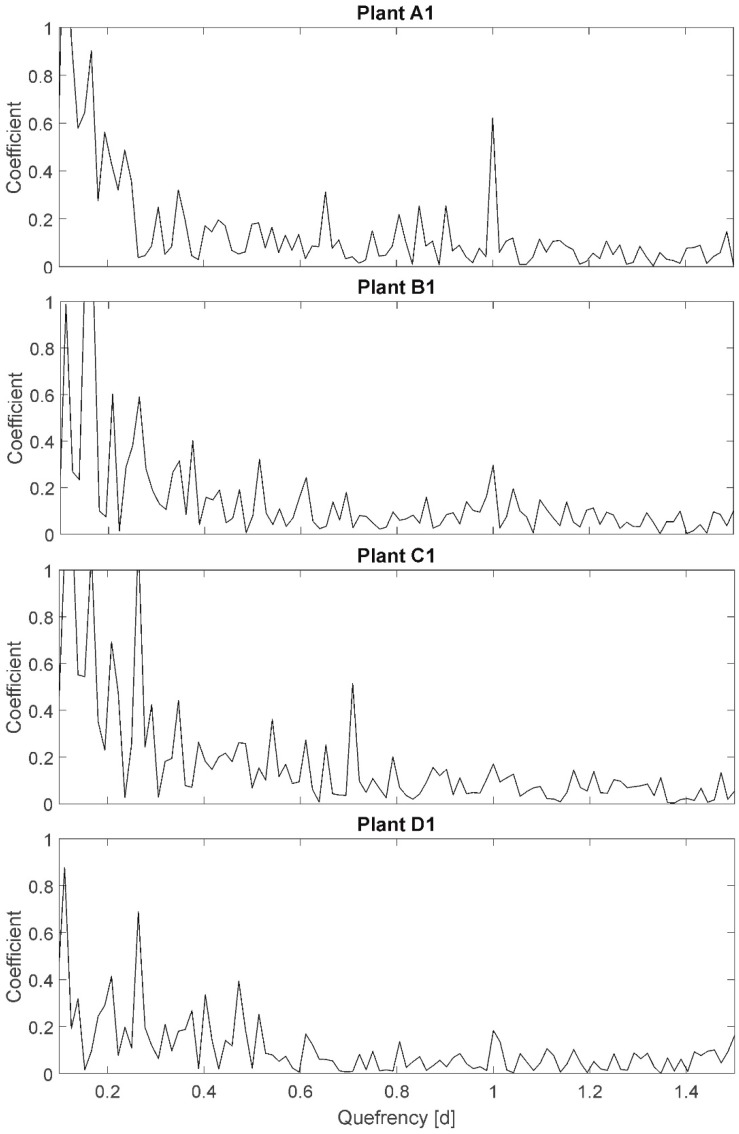
Cepstrum of plants A1, B1, C1, and D1. Quefrency represents a periodic time interval, and the coefficient is dimensionless and represents the echo of a specific frequency.

Cepstrum analysis is based on a double Fourier transform and is used to detect common sources within a mix of resonances and related sidebands within a signal. The Fourier transform is a mathematical method to decompose a signal (time series) into its different frequencies, e.g., the different frequencies of an acoustic signal. Cepstrum represents a specific mathematical application of the Fourier Transform in order to detect ‘echoes’ of a specific source characterized by a whole-numbered multiple of a basic frequency. The method is used, e.g., for vibration monitoring of machines with rotating machine parts. The reliable interpretation of a Cepstrum chart is only possible, if the value of the ‘peaks’ representing a sideband are obvious (e.g., 3-fold that of the other peaks). A higher similarity in peak values and distributions cannot be rated. By applying Cepstrum analysis repeating periodic components in a signal can be detected, even at very small amplitudes (as long as the amplitudes are equal). The resulting Cepstrum of a time series represents the time period of one period length/duration of this periodic component of the signal (cf. Figure 5).

#### 2.3.4. Short-Term Fourier Transform (STFT)

**Figure 6:**
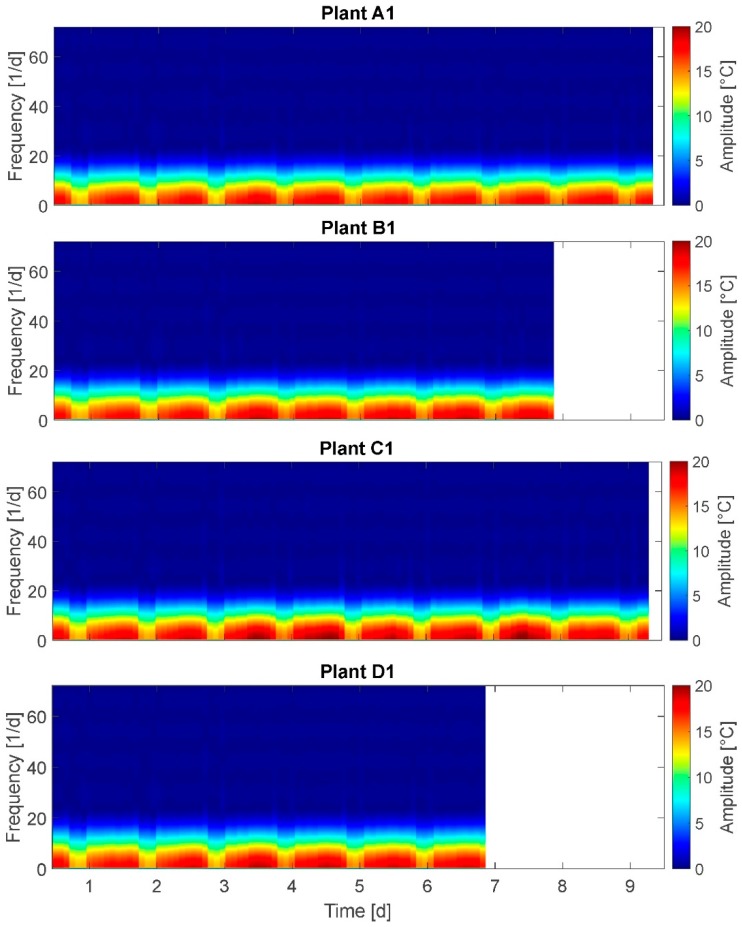
Short-term Fourier transform of plants A1, B1, C1, and D1. Time–frequency–energy representation of leaf temperature time behavior.

The STFT is also based on the Fourier transform, modified by the introduction of time windows with corresponding frequency spectrums (cf. [43]). Frequency changes in time (e.g., due to stimuli or disturbances) cannot be detected by a ‘simple’ Fourier transform, as this method is only applicable to stationary signals (without changing frequency properties). By applying STFT to a signal the temporal variation in the frequency spectrum of a signal can be represented (cf. Figure 6). Restrictively, STFT does not appear to be in all cases the best method for FA, e.g., if short-term high-frequency signal components concur with slow variant low-frequency signal components as, for example, in Electroencephalography (EEG) signals (cf. [44]; cf. Küpfmüller’s uncertainty principle, stating that a high time resolution, e.g., to detect the exact time of appearance of a certain frequency component, is not compatible with a high frequency resolution for exact frequency determination). Therefore, as the particular frequency components of the presented leaf temperature signal is unknown, the below described alternative methods are also applied to the signal.

#### 2.3.5. Wavelet Transform (WT)

**Figure 7:**
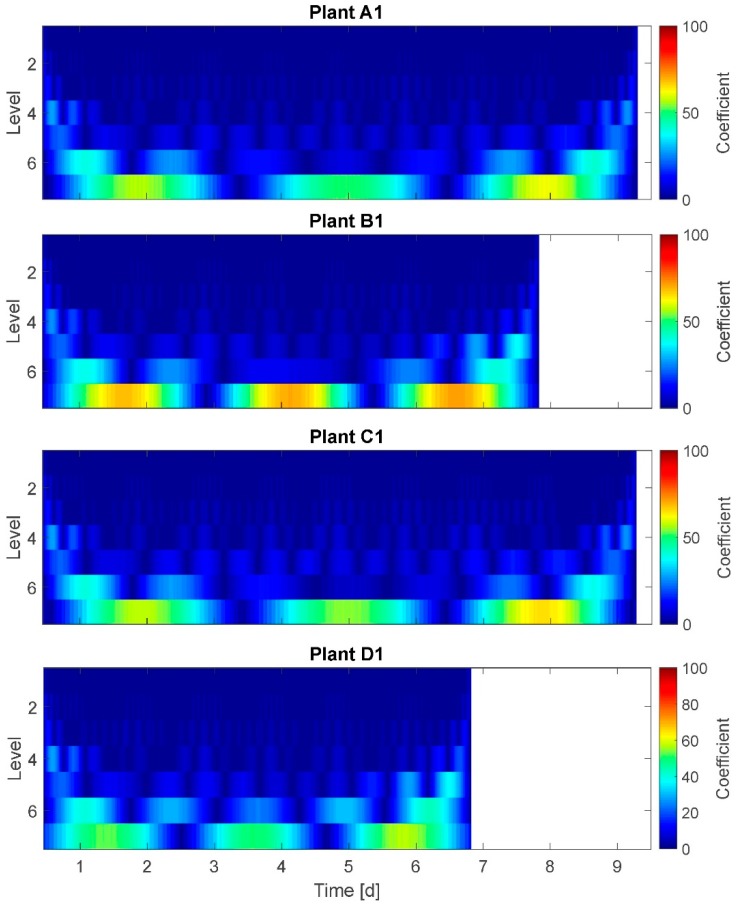
Wavelet transform of plants A1, B1, C1, and D1. For the seven model-based frequency levels the coefficient represents the amplitudes of oscillations.

The WT was developed to solve the named problem of an adequate frequency resolution for low-frequency signal components and an adequate time resolution for high frequency components of a signal. With WT the signal is separated into frequency adopted time sequences to analyze simultaneously the frequencies of different lengths in accordingly selected time windows (cf. [45]). As a drawback of WT compared to STFT, the lack of an amplitude representation in the analysis should be noted. The application of WT to a signal facilitates the frequency pattern recognition in signals, as signal components of different frequencies can be considered at the same time, although at different resolutions (cf. Figure 7).

#### 2.3.6. Stockwell Transform (ST)

**Figure 8:**
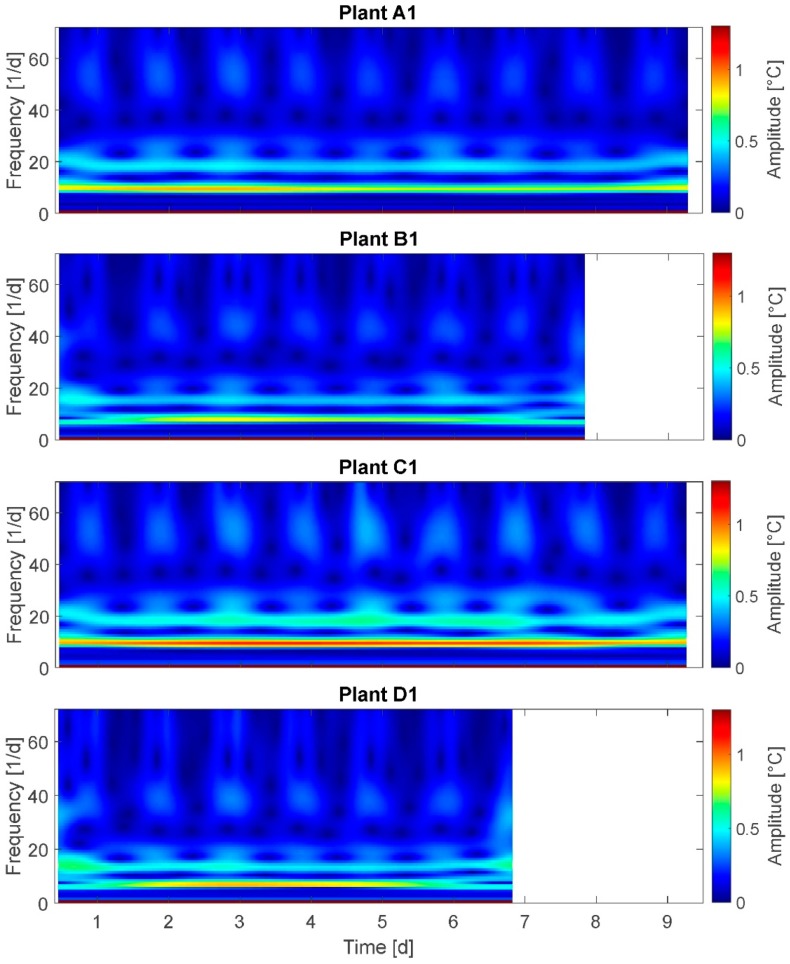
Stockwell Transform of plants A1, B1, C1, and D1. Time–frequency–energy representation of leaf temperature time behavior

The ST is a recently developed and consistently enhanced FA method. The ST is based on a modified short-term Fourier transform (STFT) method adjusting the time–frequency–resolution automatically to the relevant frequency and hence combining the strengths of STFT and the Wavelet Transform (WT), i.e., a high time resolution and a high frequency resolution (cf. [46]) (cf. Figure 8). However, also this method is not always the optimal FA method for all applications, drawbacks are, e.g., still the comparably high computational time needed to analyze big data volumes.

#### 2.3.7. Hilbert–Huang Transform (HHT)

**Figure 9:**
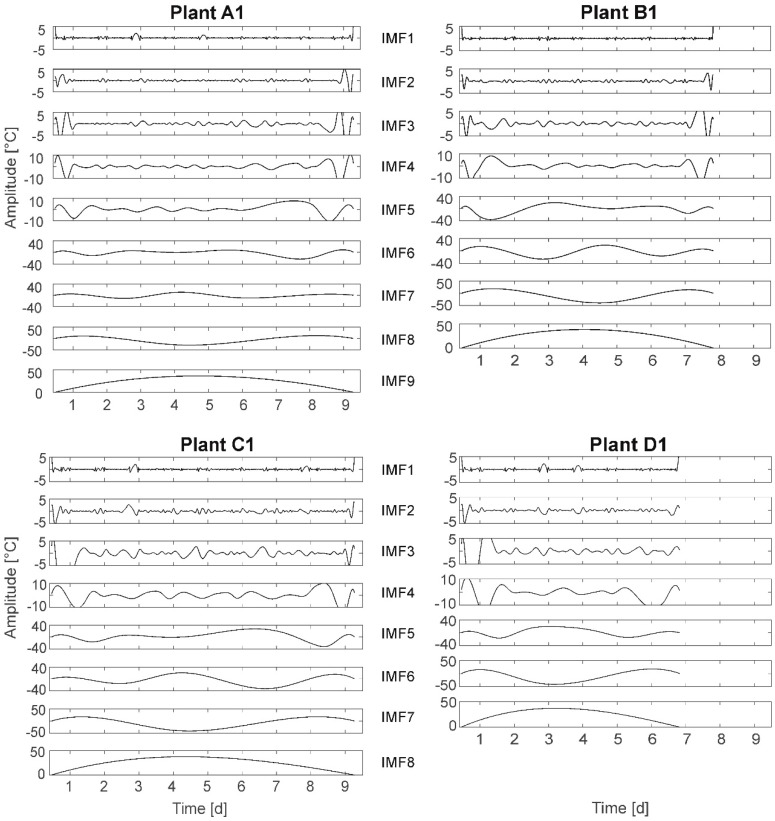
Hilbert–Huang Transform of plants A1, B1, C1, and D1. Frequency spectrum is depicted in a set of curves (IMFs) with the time on the x-axis and the amplitude on the y-axis. In HHT amplitudes of up to 50 °C are depicted. These high temperature values are not real values in terms of surrounding air temperature, but are a result of the specific algorithm used in HHT. Here, amplitudes larger than the natural temperature variations represent superimposed vibrations of the signal of lower frequencies hidden in the temperature signal, and therefore are depicted in °C.

This time–frequency data analysis method includes an empirical mode decomposition part and a Hilbert spectral analysis part. First, an iterative algorithm is fragmenting the signal into so called Intrinsic Mode Functions (IMFs) representing the individual, superimposed oscillations. Second, a Hilbert spectrum analysis is applied to the IMFs to detect the instantaneous frequencies in time (cf. Figure 9). The result represents a “physically meaningful time–frequency–energy description of a time series” [47]. Contrary to Fourier transform-based methods, the HHT is an empirical approach utilizing the structure/characteristics of measurement data to detect oscillations of different kinds within the same signal. The method is supposed to uncover concealed physical relations and to facilitate the search for disturbance-initiated natural oscillations.

All a.m. methods (except MM and PP) are tools for signal processing procedures, e.g., in control engineering, geophysics, speech recognition, or medical applications. As mentioned, each method has pros and cons (cf. Table 1).

The selection of a certain method for a specific application should be based on considerations concerning the expected period durations and frequencies, the required resolutions, computational aspects, and the target of the study. In this study, caution is required if time series of different time duration are compared, e.g., with ST, as the duration/length of the time series has influence on the graphical representation of frequencies. This drawback can be handled by unifying time series length or by using the continuous (instead of the discrete) version of WT (as done in this study). Important for the use of any of the methods for explorative purposes is the intended testing of different sets of parameter values (different resolutions, coefficients, and levels) in order first to explore the abilities of the methods for the specific application and, second, to detect potential misinterpretations because of methodological drawbacks of the methods. Finally, without any theoretical background or assumptions regarding the actual (biological) processes behind, the interpretation of FA methods can be misleading. The methods most intuitively comprehensible for persons not familiar with the mathematical background of the methods are MM and PP (as simple plot methods), and HHT (as data-based instead of theory-based method).

In this study, all named methods are applied to the data to analyze unknown features of the measured data and to explore the explanatory power of the different methods for leaf temperature behavior. For application, these methods are available in the form of toolboxes for analysis software. To perform the numerical results, the signal processing toolbox of MATLAB (Mathworks ©) was used.

## 3. Results

Derived from the above-cited studies on stomatal oscillation behavior (e.g., [26,32,34]), the expected results of the frequency analysis are as follows.

Assumption I (A-I): Water deficit results in transpiration reduction because of stomatal closure and hence in rising leaf temperatures (and leaf temperature amplitude accordingly) [1,4,48]. This can be expressed by

A_S_ > A_FI_,

with A_S_ denoting oscillation amplitude A of plants in the state “water-stressed”, and A_FI_ denoting oscillation amplitude A of plants in the state “nonwater-stressed”.

Restrictively, considering the superimposed transpiration oscillation amplitudes due to stomatal oscillations as investigated for oat plants [49], the amplitudes can show another behavior: The stepwise reduction of water potential in the root medium resulted initially (at mild stress) in a reduced transpiration oscillation amplitude, and finally (at high stress) to an immediate stop of oscillation activity. As the wavelength was not affected by the treatment in that experiment, the transpiration rate amplitude reduction is based on a reduction of stomatal conductivity, e.g., by reduced stomatal opening width. This result is in accordance with the a.m. assumption A-I, as reduced transpiration results in an expected leaf temperature rise. However, the investigation of leaf temperature frequency behavior can show superimposed oscillations with opposite amplitude behavior.

Assumption II (A-II): Water deficit results in stomatal oscillation (start condition), and hence in leaf temperature oscillation [29,32,33]. This can be expressed by

F_S_ > F_FI_.

with F_S_ denoting oscillation frequency F ([Hz] or [1/d]) of plants in the state “water-stressed”, and F_FI_ denoting oscillation frequency of plants in the state “nonwater-stressed”. Here, oscillation frequency is denoted as the number of repeated stomatal opening/closing activities in time resulting in a periodic leaf temperature signal.

As described above, this specific oscillation start behavior is not observed in all experiments of the a.m. studies. However, the application of abrupt root medium water potential reduction pulses resulted in transpiration oscillation phase shifts due to hydropassive stomatal openings [49]. Phase shift and amplitude value were altered depending on pulse duration und phase position at pulse start. Therefore, at least hydropassive openings due to water stress incipience can also result in measurable frequency alterations and can hence be detectable by standard FA methods.

### 3.1. Leaf Temperature Time Series

In Figure 2, leaf temperature time behavior of an exemplary one plant of each group (A1, B1, C1, and D1) is depicted. The presented leaf temperature time behavior is characterized by abrupt temperature changes (gray, vertical lines) when illumination is switched on/off (day–night cycle). Temperature signal gaps at these moments amount to 2.5–3 °C of the calculated temperature due to the sudden discontinuation of radiation. It is assumed that the determined difference in leaf temperature is not reflecting a real cool down of leaf temperature, but is mainly due to the radiation-based measurement technique of bolometric IR cameras. To depict real cool down processes a data processing subtracting this known “virtual” temperature gap could be applied. The presented results are based on the original, unprocessed data. Here, only temperature differences between the irrigation treatments are considered, not absolute values. Restrictively, when evaluating leaf temperature behavior under the described, not sinusoidal illumination regime, transient transpiration behaviors due to the sudden light activation/discontinuation can possibly cause transient leaf temperature behavior until leaf temperature commute in similar level as in a natural environment (including dusk and dawn). One explanation for this behavior may be a delayed response of the stomatal apparatus to external light signals (dead time) [50].

Besides, this day/night oscillation is fixed to an artificial period of 24 h, and different oscillations are detected regarding the temperature time behavior in Figure 2: oscillations of small amplitudes (<1 °C) and short periods (<1 d) in the days and partially also at nights (e.g., in solid lined circles), oscillations of higher amplitudes (1–2 °C) and longer periods (3–4 d) at days (between dashed lines), and oscillations with amplitudes of approximately 1 °C and periods up to 5 days at nights (between dotted lines). Here, the time series is considered to be “raw” data. The different frequencies are superimposed with overlapping oscillations of different period durations and amplitudes. From this it can be stated that a visual periodicity is not expected to be identified at this stage of analysis.

### 3.2. Local Minima and Maxima (MM)

In Figure 3 the results of the MM are presented. Here, the measured local maxima (attributed to stomatal opening and resulting in leaf temperature decrease) and local minima (attributed to stomatal closure and resulting in leaf temperature increase) for the plants A1, B1, C1, and D1 are depicted. These local extrema represent the inflection points at which the leaf temperatures switch from decreasing values to increasing values (or vice versa). This means only the respectively smallest measured value before a temperature rise and the respectively highest measured value before a temperature decrease are depicted in the diagram.

The connecting lines between the axis of abscissae and the particular signal values denote the events of stomatal opening/closing. Hence, the line sequences denote the oscillation rhythms: the higher the density of lines, the higher the stomatal activity (opening/closing) is. It was expected to observe a distinct change in oscillation rhythms of stressed plants compared to fully irrigated plants (cf. assumption A-II).

Selected results: Leaf temperature oscillation was measured for all plants during daytime as well as at night independent of irrigation treatment. Different leaf temperature oscillation rhythms are found as well for all plants independent of irrigation treatment. Here, rhythms are denoted as periods of very high oscillation activity (extremum/inflection point at each measurement (which can be qualitatively detected independent from sampling rates)), periods of regular and lower oscillation (about one local extremum/h), and periods of no oscillation for more than one hour (rest period). Further, leaf temperature oscillation rest periods are regularly, but not only, detected for the nights and measured for all plants independent of irrigation treatment. These rest periods often start (>50 %) before illumination is switched off.

Summarizing, the analysis of MM does not support the assumption A-II, that a water deficit is a start condition for stomatal oscillation.

### 3.3. Phase Portrait (PP)

The results of the PP are presented exemplary for the same plants A1, B1, C1, and D1 (Figure 4). On the x-axis the state variable leaf temperature and on the y-axis the first derivative (leaf temperature change rate between each measurement, Δt = 20 min) are plotted. Each measurement value is represented by a node. The nodes are connected by edges resulting in a (angular) curve.

The plotted measurements depict the oscillations by moving in a circle turning clockwise. Different colors denote different daily cycles. For example, the night temperature oscillates in small steps of ~0.1 °C decreasing for ~2 degrees per night (15.5 °C to 13.5 °C). When light is switched on, the temperature values “jump” from the lowest night value of the respective day in ~2–4 measurements (nodes) to the lowest respective daily value at ~17 °C (end node at the descending edges of the upper circles).

Two equilibria, or areas of attraction, are depicted, denoting temperature ranges during daytime (accumulation/queue of small circles, e.g., for plant A1 at temperatures between 16 °C and 19 °C) and at night (accumulation/queue of very small circles, e.g., for plant A1 at temperatures between 13.5 °C and 15.5 °C). These two equilibria for the values during daytime and the values at night are illustrated by the two smaller, broken-lined ovals in the PP of plant A1 and exemplary also for plant C1. Plant C1 (high stress) shows a shift of the equilibrium point in the days towards higher leaf temperatures. The vector field is illustrated exemplary by the large, broken-lined oval in the PP of plant A1 and C1 representing the totality of all measured leaf temperature values for the particular plant. Here, the vector field of plant C1 is extended compared to the one of plant A1. The value of the leaf temperature change rate denotes the speed of temperature changes between two measurements. It was expected to observe a vector field extension (additionally, higher leaf temperatures and change rates) for stressed plants compared to fully irrigated plants (cf. assumption A-I).

Selected results: Overall leaf temperature ranges during daytime are higher for plants in states “water-stressed” (16.0–22.0 °C) than for plants in state “nonwater-stressed”: (16.0–19.5 °C). This means, that stressed plant produce higher total leaf temperatures. But, leaf temperatures of highly water-stressed plants exceeding the maximum leaf temperature of 20 °C (maximum leaf temperature of well-watered plants) are detected only for two plants. Further, maximum plant individual heating up during daytime (lowest to highest daily temperature value is higher for plants in state “water-stressed” (1.5–4.0 °C) than for plants in state “nonwater-stressed” (1.0–2.5 °C). However, maximum plant individual heating-up during daytime of highly water-stressed plants of more than 2.5 °C (maximum value of well-watered plants) are detected only for three plants (group C). This result does not support assumption A-I unambiguously. Contrary to that, cooling down of leaf temperatures during daytime before switch-off of illumination is slightly higher for plants in state “water-stressed” (0.5–1.5 °C; two highly stressed plants of group C: 2.0 °C) than for plants in state “nonwater-stressed” (0.5–1.0 °C); this again supports assumption A-I. Overall cooling down ranges of leaf temperatures in the night range for all plants between 0.5–1.5 °C independent of irrigation treatment. In this experiment, water regime is not influencing the cooling amplitude during night.

Summarizing, the analysis of PP does not generally support the assumption A-I that a water deficit acts as a condition for stomatal oscillation amplitude rise.

### 3.4. Cepstrum (CEP)

The results of the CEP are exemplary presented for plants A1, B1, C1, and D1 (Figure 5). On the x-axis, the so-called quefrency is plotted, representing a periodic time interval of redundant signal components. On the y-axis, a dimensionless coefficient, representing the existence/nonexistence of side bands (periodic components) of individual frequencies, is plotted. For example, the quefrency of 1.0 corresponds to the period of 1 d (24 h). In the plot of plant A1 a peak with the y-value of 0.6 is shown at a quefrency of 1 d. This represents the 24-h light–darkness rhythm in this experiment. The peak is not as conspicuous as expected, knowing that the 24-h side band is artificial and very precise in period duration. This is due to the plant individual diurnal variations in leaf temperature amplitudes (cf. Figure 2: e.g., oscillation at a period duration of 3–4 days with amplitudes of 1–2 °C in the days). The CEP method only considers quefrencies of similar amplitudes being part of a side band. Whereas, well-watered plants, like A1, still show a significant peak at quefrency 1.0 (approximately 3-fold of the neighboring values), all other plants do not show the 24-h side band because of the variation in diurnal temperature amplitudes despite the precisely equal period duration.

Amplitudes are not particularly plotted in CEP, but as only repetitive amplitudes of similar values are considered to be part of the side band, a variance in amplitudes is detectable by a nonexistence of considerable peaks. Here, “considerable” is only defined qualitatively: “considerable” denotes, that the peak has to be more than double to three times higher than the ambient peaks in order to be significant. Here, according to the Shannon–Nyquist Theorem all quefrencies higher than 0.03 are reliable (cutting edge frequency of 36/24 h). A variation of the quefrency resolution (e.g., towards higher quefrencies than 1.5) did not show particular results. This could be different if the experiments duration would be extended to detect oscillations of longer period durations. The large number of high peaks at quefrencies below 0.3 partially result from an ‘unclean’ resolution at these quefrencies. A variation of resolution towards smaller quefrencies did not show significant results.

However, it was expected to observe a distinct sideband displacement for stressed plants compared to fully irrigated plants representing higher frequencies.

Selected results: The peak at quefrency 1.0 represents the known day–night rhythm, which is considerable for plants in group A (well-watered, all plants peak > 0.4), less significant for plants in group B (mild stress, all plants 0.2 < peak < 0.4), and even less significant for plants in group C (high stress, all plants peak < 0.2). This can be interpreted as an increasing difference in amplitude in time resulting from leaf temperature increases for stressed plants (cf. PP). On the other hand, plants in group D (not at all irrigated) show peak values at a quefrency of 1.0 between 1.8 (D1, very uniform amplitude) and 0.4 (D2, D3; comparable to group B). The result is therefore not unambiguous related to assumption A-I. Further, the peak at quefrency 0.25 represents a periodic frequency component every 6 hours: ~60 percent of all plants independent of irrigation treatment have a peak higher than 0.6 at this quefrency (in Figure 5 only represented for plants B1, C1, and D1). The ambient peaks are also comparatively high; therefore, this peak is assumed to be of no relevance. However, as it relates to all plants, irrigation treatment is not relevant for this sideband and also not for assumption A-I.

Additional peaks with coefficients higher that 0.4 are detectable at quefrency 0.5 for plant B2 and B5 (mild stress), and at quefrency 0.7 for plant C1 (high stress), indicating periodic components at that period duration. The information cannot support assumption A-II (sideband displacement due to higher or additional frequencies), as no additional peaks for all other plants can be detected.

Summarizing, the results of the CEP analysis do not unambiguously support the assumptions.

### 3.5. Short-Term Fourier Transform (STFT)

The results of the STFT are presented for plants A1, B1, C1, and D1 (Figure 6).

On the x-axis the time is plotted and on the y-axis the frequency is plotted. The spectrum at the right side denotes the amplitudes of oscillations. Here, amplitudes of up to 21 °C are depicted, representing the total, measured leaf temperatures. It was expected to observe a distinct frequency spectrum change for stressed plants compared to fully irrigated plants.

Applying STFT the day–night cycle can be clearly depicted with regular amplitude alternations of approximately 16–20 °C, which is in accordance with the PP results. At frequencies from 1–5/24 h the amplitude exceeds 18 °C for plants A1, B1, and D1, but only at middays, while for plant C1 frequency and duration of maximal amplitude are higher. A water deficit-based behavioral change expected at frequencies of 24/24 h and more could not be detected.

Selected results: Nonwater-stressed plants show amplitudes exceeding 18 °C at frequencies of 1–5/24 h only at noon, whereas water-stressed plants show these higher leaf temperature amplitudes also in the mornings. This supports assumption A-I. On the other hand, only in one group (C) this behavior can be detected. This limits the significance of the result. Further, no difference in frequencies for amplitudes between 1 °C and 20 °C is observed for all plants irrespective of irrigation treatment.

Summarizing, the STFT analysis also does not support the assumptions.

### 3.6. Wavelet Transform (WT)

The results of the WT are presented for plants A1, B1, C1, and D1 (Figure 7). On the x-axis time is plotted and on the y-axis the model-based frequency levels are plotted. Here, seven frequency levels are given. The spectrum at the right side denotes a coefficient representing the amplitudes of oscillations. Although the coefficient scale of 0–100 does not represent real amplitudes, high coefficient values are attributed also to high amplitudes. A direct conversion of the coefficient into amplitudes is not possible.

In the graph ‘time blocks’ of approximately equal time durations are depicted at each frequency level: Here, the width of the blocks/segments of a higher frequency level (e.g., level 4) are an integer multiple of the width of the next lower frequency level (level 5, correspondingly). This represents the model-based modified time resolution on behalf of a higher frequency resolution in the respective level. The ranges of time are determined by the WT according to the given data and the number of levels chosen. Therefore, the day–night cycle is not exactly met at any of the levels but located between levels 5 and 6. The adjustment options for WT diagrams relate to the (amplitude-related) coefficient and the number of levels (time resolutions). Therefore, the interpretation of WT for this application is not as comprehensible as, e.g., of the STFT.

In the shown WT result, each frequency level is textured in approximatively symmetric, regular amplitude segments of time durations between 2 h (level 2) and 72 h (3 days) (level 7) with

- amplitude coefficient of up to 30 on the higher frequency levels 1 to 5,
- amplitude coefficient of up to 55 on the frequency level 6, and
- amplitude coefficient of up to 100 on the lowest frequency level 7.

This means for the example of plant C1 that the segments on level 6 have approximately the 2.5-fold duration of the segments on level 5, and the segments on level 5 have approximately the 1.25-fold duration of the segments on level 4. Here, plant B1 is an exception: at frequency level 6 plant B1 has one nearly double-sized segment between day 3 and day 5. This indicates a higher amplitude coefficient at day 4 compared to other days (and plants). Conspicuous changes in frequency or amplitudes at moments in time for which it is known that plants experienced water stress cannot be detected.

It was expected to observe a distinct frequency spectrum change for stressed plants compared to fully irrigated plants. Selected results: At level 6 (time window width: 1.5 days) the amplitude coefficient of highly stressed plants is higher (55–60) compared to well-watered or mildly stressed plants (~40). This supports the assumption of higher amplitudes at least for highly stressed plants. On the other hand, the amplitude coefficient is maximal for all plants at level 7 (60–100), but the plants in the group without any irrigation reached just the coefficient level of the mildly stressed plants, or even well-watered ones. This does not support assumption A-I. Summarizing, based on the WT graphs, no distinct frequency spectrum can be identified.

### 3.7. Stockwell Transform (ST)

The results of the ST are presented for plants A1, B1, C1, and D1 (Figure 8). On the x-axis the time is plotted and on the y-axis the frequency is plotted. Maximum frequency is 72/24 h, equaling the sampling rate. The spectrum at the right side represents the amplitudes of oscillations.

Here, the small amplitudes of 0.0 to 1.2 °C are selected according to the specific ability of ST to analyze higher frequencies. This is also the reason why the day–night cycle is not detectable: As the leaf temperature amplitude changes due to the light-on/light-of cycle amounts to 3–4 °C amplitudes these events are not represented in this diagram. According to the Nyquist–Shannon Theorem, only the measured frequencies below 36/24 h are considered in the following observations. It was expected to observe a distinct frequency spectrum change for stressed plants compared to fully irrigated plants.

In ST continuous amplitudes and alternating amplitudes can be detected. For example, at a frequency of 8–10/24 h a continuous amplitude of approximately 0.8 °C for groups A, B, and D, and of approximately 1.2 °C for group C is depicted. This is in accordance with the observation that the maximum daily amplitudes are observed in group C. Further, at frequency 18–22/24 h an alternating amplitude between the values of 0.1 and 0.5 °C in a daily rhythm is observable for all plants. Deduced from the above-cited studies on water deficit-related stomatal oscillations, a water deficit-based behavioral change would be expected at frequencies of 24/24h and more. This could not be observed for any of the plants in this experiment.

Selected results: Amplitudes at frequencies of 8–10/24 h are 50 % higher (up to 1.2 °C) for highly stressed plants compared to nonwater-stressed ones. On the other hand, this is only observed for 50 % of the highly stressed plants. This does not support the assumption A-I unambiguously. Further, the expected higher frequencies at amplitudes of 0.1 to 1.2 °C for highly stressed plants could not be observed, also not for any plant irrespective of irrigation treatment. This does not support assumption A-II.

Summarizing, the results of ST analysis do not support assumption A-II; and assumption A-I is only partly supported.

### 3.8. Hilbert–Huang Transform (HHT)

The results of the HHT are presented for plants A1, B1, C1, and D1 (Figure 9). The frequency spectrum is depicted in a set of curves, so-called IMFs. For each IMF (frequency) on the x-axis the time and on the y-axis the amplitude are plotted. The lowest IMF in each case defines the stop function of the algorithm and does not represent a frequency. The stop function can be selected from a set of options. Here, the so-called S number criterion [51] is chosen, stopping the algorithm if for a number of consecutive siftings (loops) the number of extrema and zero-crossings stay the same (or plus one). It was expected to observe a higher number of IMFs and/or IMF time behavior changes for stressed plants compared to fully irrigated plants.

In HHT the total number of IMFs per plant represents the number of different frequencies identified in the leaf temperature time behavior as

- group A (FI): two plants have nine IMFs, three plants have eight IMFs;
- group B (MS): five plants have eight IMFs;
- group C (HS): two plants have seven IMFs, three plants have eight IMFs; and
- group D (NI): three plants have seven IMFs, two plants have eight IMFs.

The general conclusion, that water deficit results in a smaller number of IMFs cannot be drawn, as plants with seven IMFs also died earlier in the experiment, resulting in a shorter time series. However, plant A1 (and A3, not shown here) has nine IMFs compared to other plants of equal life time and only eight IMFs. Here, one additional frequency is detected.

Further, HHT based on the total time series (10 days) results in two more IMFs than HHT based on split single-day time series composed to the 10-day period (not shown here). This indicates superimposed multiday frequencies.

Analyzing the different IMFs following regularities can be determined: In IMF1 the light–darkness oscillation at amplitudes below 5 °C can be detected for all plants. In IMF2 an obviously day–night-based oscillation at amplitudes below 5 °C can be detected. This oscillation is not exactly aligned to the light–darkness cycle. The related behavior can be observed for all plants. In IMF3 and IMF4 (for A1 and A3 also partly in IMF5) oscillations of frequencies higher than 1/24 h are depicted. The amplitudes are below 10 °C (IMF3) and below 20 °C (IMF4, for A1; A3: IMF5)). In IMF5, IMF6, and IMF7 (A1 and A3: IMF6, IMF7, and IMF8) oscillations show frequencies of less than 1/24 h (multiday frequencies). The amplitudes are given with up to 40 °C. The maximum leaf temperatures are obviously depicted in IMF6 (A1/A3: IMF7). For example, plant C1: maximum leaf temperatures appear at days 4/5 and 8 (cf. Figure 2). This can be detected in IMF6. It should be noted that in HHT amplitudes of up to 50 °C are depicted. These high temperature values are not real values in terms of surrounding air temperature, but are a result of the specific algorithm used in HHT. Here, amplitudes larger than the natural temperature variations represent superimposed vibrations of the signal of lower frequencies hidden in the temperature signal and therefore depicted in °C.

Comparing the HHT of the four plants with different irrigation treatments the symmetry in IMF5 is remarkably different: The fully irrigated plant (A1) has a plateau of about 20 °C amplitude between the days 2 and 6. This plateau is compressed to an elevation for the nonirrigated plant (D1), as well at amplitude of 20 °C, probably because of the shorter lifetime. This symmetry is not present for plants B1 (MS) (elevation on the left: earlier, higher amplitude up to 20 °C) and C1 (HS) (elevation on the right: later, higher amplitude up to 20 °C). This observation cannot be ascertained for all plants in each group, but the number of symmetric IMF5 (A1/A3 IMF6) is highest in group A (3), lowest in group C (1), and IMF5 in group B is only symmetric or with an elevation on the left whereas IMF5 in group C is only symmetric or with an elevation on the right. On the other hand, elevations on both sides, right or left, are both found also in the groups A (FI) and D (NI).

Selected results: Contrary to assumption A-II, the number of IMFs is rather higher for fully irrigated plants then for stressed plants. This is not only due to a longer lifetime, as also plants from groups B and C survived the complete experiment. Further, a distinct shift/change in IMF time behavior for stressed plants also cannot be unambiguously detected, just as an increase in amplitudes. This result does not support assumption A-I. Finally, at least one of the detected frequencies (day–night cycle) is not plant-based.

Summarizing, the results of HHT do not support the assumptions. A further investigation of the reason for the appearance of the different IMFs is recommended.

### 3.9. Results Overview

The above-described findings of the different explorative frequency analysis methods are recapped in Table 2. The results do not unambiguously support the assumptions, that leaf temperature amplitude (A-I) and frequency (A-II) of plants in water-stressed states are generally higher than those of plants in well-watered states.

## 4. Discussion

These at first sight unsatisfactory result retrieves a number of interesting observations and questions:

### 4.1. Leaf Temperature Variability

A higher amplitude (A-I) for stressed plants was detected, but not for all stressed plants. One reason for this result can be, that the variability of leaf temperatures is already high (3 °C, 16.5–19.5 °C) between all plants at well-watered state at the beginning of the experiment (day 1). As the experiment progressed, the variability of well-watered plants remains between 2.0–2.5 °C (16.5–19.0 °C in the days) during the entire experiment, whereas the variability in mildly stressed plants (groups B, C, and D at day 1.5–2.5) increases to 3.5 °C (16.5–20 °C in the days), and in highly stressed plants (groups C and D at day 2.5–3.5) to 5.0 °C (17.0–22.0 °C in the days). However, only three highly stressed plants of groups C and D exceeded the maximum variability value of 2.5 °C (maximum value of well-watered plants). Restrictively, the generalization of single-point leaf temperature measurements to whole leaf temperatures and hence to whole leaf transpiration is not possible, as stomata do not show in all cases synchronized behavior [52]. However, based on the assumed causality between plant water stress and transpiration rate/leaf temperature and as ~1890 single measurements of highly stressed plants were taken during this experiment, the rate of highly stressed plants with distinct leaf temperature elevations could be expected to be higher than 30%. This means in practice that under constant and equal environmental conditions one objectively well-watered plant with a leaf temperature of 17.5 °C is compared to an objectively highly stressed plant of equally 17.5 °C leaf temperature. The related research question is as follows. Is leaf temperature per se representing the actual plant water supply status? Or in detail: What is the detailed connection between the dynamical character of leaf temperature signal and water supply status? This question has implications on the use of leaf temperature signals for irrigation scheduling: Based on the presented results, plant individual absolute leaf temperature signals per se cannot be used for reliable irrigation scheduling. Instead, variability parameter of leaf temperature signals of canopies can serve as indicator for a general stress incipience. Here, stress-sensitive plants indicate water stress for all plants of a crop. This interpretation, of course, is restricted based on the presented results only to homogenous environmental conditions. However, the use of leaf temperature variability has been proposed based on field experiments [53].

### 4.2. Leaf Temperature Maintenance

The largest plant-individually measured daily temperature rise under the presented conditions is 4 °C (plant C3, day 3, state high water stress). This represents the potential minimum leaf temperature increase all highly stressed plants could physically have experienced. The smallest plant-individually measured daily temperature rise of objectively highly stressed plants under the presented conditions is 2 °C (plant D4, day 5, state high water stress).

This value is similar to the plant-individually measured daily temperature rise of well-watered (1.5–2.5 °C) or mildly stressed plants (1.5–2.0 °C). The resulting question is how is a highly stressed plant able to maintain leaf temperature values comparable to those of well-watered ones? This result could be caused by open stomata despite wilting and minimum leaf water potential in stressed plants [54]. Further, according to Laleh et al. [55], additional aspects despite pure hydraulic conductance considerations can also have an effect on plant water balance and hence transpiration and leaf temperature behavior.

### 4.3. Leaf Temperature Oscillation Start Condition

A higher leaf temperature oscillation frequency of plants in water-stressed states (A-II) was not detected. Hence, the start condition “water deficit” for an expected additional stomatal oscillation activity could not be measured under the presented experimental conditions. As desiccation is a continuous process, at least at in some (mild) states of water deficit a frequency change was expected to occur, even if stomatal oscillation would stop in the course of further dehydration (high stress). This assumption is also generally supported by the study of Cowan [56], showing the occurrence of leaf conductance oscillations due to step changes in potential transpiration, root resistance, or base water potential. Here, initial small step changes result in attenuated oscillations and increased step changes result in perpetuated oscillations. This behavior was not observed in this study. Potential reasons for this are listed as follows.

- Synchronized stomata: Oscillation of transpiration and hence oscillation of leaf temperature can only be detected if stomata behave in a synchronized manner [30]. This also applies to changes in oscillation behavior. An undetected change in oscillation frequency could therefore also result from unsynchronized stomatal behavior. However, this explanation would be in contradiction to the above mentioned assumption of a synchronized hydraulic signal controlling stomatal behavior.
- Sampling rate: At a sampling rate of 72/24 h, oscillations up to a theoretical maximum cut-off frequency of 36/24 h can be detected. This represents the expected water deficit induced oscillation frequency described in literature. If the frequency induced by a water deficit is actually, significantly, and regularly higher than 36/24 h, the sampling rate of the existing measurements does not allow to detect variations of higher frequencies. On the other hand, the probability is small that 14400 measuring points (20 plants, 10 days, 72/24 h sampling rate) is not sufficient to detect any changes in frequencies, as this would implicate that invariably all measuring points were taken in striking distance to the zero baseline of the water deficit induced oscillation. This is unlikely. However, further experiments are recommended utilizing higher sampling rates as well as a larger sample size to improve the data base in general. Further, total duration of the experiment must generally be long enough to detect oscillations of corresponding period lengths. This is accomplished in this study.
- Causality transpiration to leaf temperature: Water deficit-related stomatal oscillation does not necessarily result directly in leaf temperature oscillation. The causality between stomatal behavior under water deficit and leaf temperature behavior is based on the assumption, that stomatal opening results immediately in a leaf temperature decrease, and stomatal closing in a leaf temperature rise. In case this instantaneously resulting leaf temperature oscillation is damped or suppressed, e.g., by evaporation processes inside the leaf without direct transpiration effects (cf. [57]), or additional cooling for example by IR radiation (cf. [58]), or influences of thermoregulation (cf. [59]), the expected leaf temperature oscillation is not directly detectable.
- Measuring mistakes and measurement noise: In principle, this may reduce informational value of the presented leaf temperature signals. Subsequent experiments (not yet completely evaluated) show similar results. Therefore, the measurements are assumed as not to be systematically wrong. Further, by applying standard frequency analysis methods the (always existing) measurement noise is systematically isolated/detectable. However, results still have to be validated and statistically ascertained.

Additional studies are necessary to ascertain and to further specify the characteristics of plant leaf temperature behavior; for example, based on the measured leaf temperature time series the HHT results in 6–8 superimposed oscillation frequencies per plant. The attribution of the single frequencies to physiological processes has to be further analyzed. Here, the distinction between plant system-based frequencies (e.g., autonomous stomatal oscillations) and environmentally induced frequencies (e.g., artificial day–night cycle) should be particularly considered.

Based on the presented results, a single plant-based identification of water stress based on the assumption of a water passive regulation of leaf temperature is not possible. The remarkable variability of the plants individual ability to regulate leaf temperature under severe water stress implicates considerably different water stress behaviors of plants within one cultivar.

However, the application of FA methods, particularly the Phase Portrait, the Hilbert–Huang-Transform, and the Stockwell Transform provide meaningful insights for the analysis of the presented data. Hence, the presented methods are generally applicable to data of this kind and origin. The selection of an adequate method should be based on considerations about on the expected period lengths and frequencies, the required resolutions, computational aspects, and the target of the study. Important for the use of any of the methods for explorative purposes is the intended test of different sets of parameter values (different resolutions, coefficients, levels) in order first to explore the abilities of the methods for the specific application and, second, to detect potential misinterpretations because of methodological drawbacks of the methods. Finally, for interpreting FA results theoretical background knowledge regarding the actual (biological) processes behind or hypothesis-based experiment design is required.

## 5. Conclusions

Application of standard frequency analysis methods to botanical data like the tested leaf temperature data provide meaningful insights into plant behavioral patterns. Known behaviors (e.g., day–night cycle) can be represented by these methods in different forms like time–frequency representations, sidebands, or intrinsic mode functions (IMFs)). Further, new information on the data can be produced like oscillation equilibrium points or the differentiation of superimposed frequencies. None of the methods generally outperforms the other as each method has pros and cons (cf. Table 1). Regarding the use in botanical scenarios particularly the Phase Portrait and the Hilbert–Huang-Transform are also accessible to nonspecialists in control engineering and allow the analysis of hidden dynamics. However, the results of the tested leaf temperature data did not support unambiguously the assumptions of a water deficit induced leaf temperature oscillation behavior for maize. Further studies are recommended to revise these results for other species and conditions.

## Figures and Tables

**Table 1:** Overview of frequency analysis methods applied in this study. Abbreviations: Local Minima and Maxima (MM), Phase Portrait (PP), Cepstrum (CEP), Short-Term Fourier Transform (STFT), Wavelet Transform (WT), Stockwell Transform (ST), Hilbert–Huang Transform (HHT), Frequency Analysis (FA).

Method	Target	Pros	Cons
MM	Visualize oscillation rhythms	Easily plotted, first insight into regularities	Qualitative, not a FA method in a narrow sense
PP	Visualize regularities of dynamic behavior	Easily plotted, first insights into dynamics	Qualitative, not a FA method in a narrow sense
CEP	Detect sideband	Detection of periodic signal components	Only frequencies of same amplitude are shown as sideband, qualitative ‘peak’ interpretation
STFT	Detect transient variations in frequency spectrum	Approved standard tool for FA in different applications	Restricted resolution for time or frequency, not optimal for signals of different time and frequency spectra within the same signal
WT	Analyze frequencies of different lengths for general signal pattern recognition	Simultaneous representation of different frequency spectra and time resolutions	Limited representation of amplitudes, discrete WT: difficult comparison of time series of different lengths
ST	Detect transient variations in frequency spectrum	Enhanced STFT method: combination of high time with high frequency resolution plus amplitudes	High computational requirements for big data volumes, comparably new method (applications are still tested), comparison of time series of different lengths
HHT	Detect concealed physical relations/disturbance-initiated natural oscillations	Distinction of different frequencies into time series, comprehensible interpretation	Not theory-based (empirical), comparably new method (applications are still tested)

**Table 2:** Overview of frequency analysis results for leaf temperature (LT) measurements of water-stressed corn plants compared to full-irrigated plants.

Method	Expected Result	Frequency	Amplitude	Observations	A-I	A-II
**Local Minima and Maxima**	Change in oscillation rhythms	Not specified	12–21 °C	All plants showed oscillations. All plants showed rest periods. Oscillation rhythms not higher for stressed plants	Not tested	Not supported
**Phase Portrait**	Vector field expansion	Not specified	12–21 °C	Stressed plants (30%) showed higher max. LT. Stressed plants (15%) showed higher daily LT-warming in the days. All plants showed equal nocturnal cooldown. Stressed plants (50%) showed higher prenocturnal cooldown.	Not unique	Not tested
**Cepstrum**	Sideband displacement	0–1.5 (36 h) (sideband range)	All amplitudes	Stressed plants (75%) showed less distinct peak at quefrency 1.0. No other distinct difference in quefrency shape Stressed plants (40%) showed additional peaks.	Not unique	Not unique
**Short-Term Fourier Transform**	Transient effects in frequency spectrum	0–72/24 h	0–20 °C	Stressed plants (30%) showed higher amplitude at frequency 1–5/d. No frequency spectrum change for stressed plants	Not unique	Not supported
**Wavelet Transform**	Transient effects in frequency spectrum	7 levels	Coefficient 100	Stressed plants (25%) showed higher amplitude coefficient at level 7. Stressed plants (50%) showed higher amplitude coefficient at level 6. No frequency spectrum change for stressed plants	Not unique	Not supported
**Stockwell Transform**	Transient effects in frequency spectrum	0–72/24 h	0–1.4 °C	Stressed plants (15%) showed higher amplitude at frequency 8–10/d. No frequency spectrum change for stressed plants	Not unique	Not supported
**Hilbert–Huang Transform**	Higher IMF number, IMF course changes	7–9 IMFs	0–50 °C *	Number of IMFs not higher for stressed plants No distinct IMF course change for stressed plants	Not supported	Not supported

* In HHT amplitudes of up to 50 °C are depicted. These high temperature values are not real values in terms of surrounding air temperature, but are a result of the specific algorithm used in HHT. Here, amplitudes larger than the natural temperature variations represent superimposed vibrations of the signal of lower frequencies hidden in the temperature signal and therefore depicted in °C. Assumption I (A-I): Amplitude of stressed plants (A_S_) is higher than of full-irrigated plants (A_FI_). Assumption II (A-II): Frequency of stressed plants (F_S_) is higher than of full-irrigated plants (F_FI_).

## References

[1] Jackson R.D., Idso S.B., Reginato R.J., Pinter P.J. (1981). Canopy temperature as a crop water stress indicator. Water Resour. Res..

[2] Leinonen I., Grant O.M., Tagliavia C.P., Chaves M.M., Jones H.G. (2006). Estimating stomatal conductance with thermal imagery. Plant Cell Environ..

[3] Wang X., Yang W., Wheaton A., Cooley N., Moran B. (2010). Automated canopy temperature estimation via infrared thermography: A first step towards automated plant water stress monitoring. Comput. Electron. Agric..

[4] Maes W.H., Steppe K. (2012). Estimating evapotranspiration and drought stress with ground-based thermal remote sensing in agriculture: A review. J. Exp. Bot..

[5] Gates D.M. (1968). Transpiration and Leaf Temperature. Ann. Rev. Plant Physiol..

[6] Janka E., Körner O., Rosenqvist E., Ottosen C.O. (2016). A coupled model of leaf photosynthesis, stomatal conductance, and leaf energy balance for chrysanthemum (Dendranthema grandiflora). Comput. Electron. Agric..

[7] Hsiao T. (1973). Plant Responses to Water Stress. Ann. Rev. Plant Physiol..

[8] Damour G., Simonneau T., Cochard H., Urban L. (2010). An overview of models of stomatal conductance at the leaf level. Plant Cell Environ..

[9] Monteith J.L. (1981). Evaporation and surface temperature. Q. J. R. Meteorol. Soc..

[10] Mahan J.R., Upchurch D.R. (1988). Maintenance of Constant Leaf Temperature by Plants—I. Hypothesis Limited Homeothermy. Environ. Exp. Bot..

[11] Upchurch D.R., Mahan J.R. (1988). Maintenance of Constant Leaf Temperature by Plants—II. Experimental Observations in Cotton. Environ. Exp. Bot..

[12] Schymanski S.J., Or D., Zwieniecki M. (2013). Stomatal Control and Leaf Thermal and Hydraulic Capacitances under Rapid Environmental Fluctuations. PLoS ONE.

[13] Jones H., Tardieu F. (1998). Modelling water relations of horticultural crops: A review. Sci. Hortic..

[14] Thornley J.H.M. (1996). Modelling Water in Crops and Plant Ecosystems. Ann. Bot..

[15] Brogardh T., Johnsson A. (1973). Oscillatory Transpiration and Water Uptake of Avena Plants. II Effects of Deformation of Xylem Vessels. Physiol. Plant..

[16] Prytz G., Futsaether C.M., Johnsson A. (2003). Self-sustained oscillation in plant water regulation: Induction of bifurcations and anomalous rythmicity. New Phytol..

[17] Tardieu F., Simonneau T., Parent B. (2015). Modelling the coordination of the controls of stomatal aperture, transpiration, leaf growth, and abscisic acid: Update and extension of the Tardieu-Davies model. J. Exp. Bot..

[18] Lösch R. (2001). Wasserhaushalt der Pflanzen.

[19] Allen R.G., Pereira L.S., Raes D., Smith M. (1998). Crop Evapotranspiration–Guidelines for Computing Crop Water Requirements.

[20] McAusland L., Vialet-Chabrand S.R.M., Matthews J.S.A., Lawson T., Mancuso S., Shabala S. (2015). Spatial and Temporal Responses in Stomatal Behaviour, Photosynthesis and Implications for Water-Use Efficiency. Rhythm in Plants, Dynamic Responses in a Dynamic Environment.

[21] Webb A.A.R., Lumsden P., Millar A. (1998). Stomatal Rhythms, in: Biological Rhythms and Photoperiodism in Plants.

[22] Hubbard K.E., Webb A.A.R., Mancuso S., Shabala S. (2015). Circadian Rhythms in Stomata: Physiological and Molecular Aspects. Rhythm in Plants, Dynamic Responses in a Dynamic Environment.

[23] Davies W.J., Gill K., Halliday G. (1978). The Influence of Wind on the Behaviour of Stomata of Photosynthetic Stems of *Cytisus scoparius* (L.) Link. Ann. Bot..

[24] Garcia-Mata C., Lamattina L. (2007). Abscisic acid (ABA) inhibits light-induced stomatal opening through calcium- and nitric oxide-mediated signaling pathways. Nitric Oxide.

[25] Araujo W.L., Fernie A.R., Nunes-Nesi A. (2011). Control of stomatal aperture: A renaissance of the old guard. Plant Signal. Behav..

[26] Barrs H.D. (1971). Cyclic variations in stomatal aperture, transpiration, and leaf water potential under constant environmental conditions. Ann. Rev. Plant Physiol..

[27] Upadhyaya S.K., Rand R.H., Cooke J.R. (1988). Role of Stomatal Oscillations on Transpiration, Assimilation and Water-Use Efficiency of Plants. Ecol. Model..

[28] Shabala S., Baluska F., Mancuso S., Volkmann D. (2006). Oscillations in Plants. Communication in Plants, Neuronal Aspects of Plant Life.

[29] Wallach R., Da-Costa N., Raviv M., Moshelion M. (2010). Development of synchronized, autonomous, and selfregulated oscillations in transpiration rate of a whole tomato plant under water stress. J. Exp. Bot..

[30] Johnsson A., Mancuso S., Shabala S. (2015). Oscillations in Plant Transpiration. Rhythm in Plants, Dynamic Responses in a Dynamic Environment.

[31] Caldeira C.F., Bosio M., Parent B., Jeanguenin L., Chaumont F., Tardieu F. (2014). A Hydraulic Model Is Compatible with Rapid Changes in Leaf Elongation under Fluctuating Evaporative Demand and Soil Water Status. Plant Physiol..

[32] Apel P. (1967). Über rhythmisch verlaufende Änderungen in der CO_2_ Aufnahme von Blättern. Bericht der deutschen Botanischen Gesellschaft.

[33] Zimmermann U., Rüger S., Shapira O. (2009). Effects of environmental parameters and irrigation on the turgor pressure of banana plants measured using the non-invasive, online monitoring leaf patch clamp pressure probe. Plant Biol..

[34] Marenco R.A., Siebke K., Farquhar G.D., Ball M.C. (2006). Hydraulically based stomatal oscillations and stomatal patchiness in Gossypium hirsutum. Funct. Plant Biol..

[35] Raschke K. (1970). Stomatal Responses to Pressure Changes and Interruptions in the Water Supply of Detached Leaves of *Zea mays* L.. Plant Physiol..

[36] Eifler W., Schlücker E., Spicher U., Will G. (2009). Küttner Kolbenmaschinen.

[37] Meade M.O., Young D., Hanna S., Zhou Q., Bachman T.E., Bollen C., Slutsky A.S., Lamb S.E., Adhikari N.K., Mentzelopoulos S.D. (2017). Severity of Hypoxemia and Effect of High Frequency Oscillatory Ventilation in ARDS. Am. J. Respir. Crit. Care Med..

[38] Gonzalez-Dugo V., Zarco-Tejadaa P.J., Fereres E. (2014). Applicability and limitations of using the crop water stress index as an indicator of water deficits in citrus orchards. Agric. For. Meteorol..

[39] Raschke K. (1965). Die Stomata als Glieder eines schwingungsfähigen CO_2_-Regelsystems: Experimenteller Nachweis an Zea mays L.. Zeitschrift für Naturforschung.

[40] Buchanan B.B., Gruissem W., Jones R.L. (2015). Biochemistry & Molecular Biology of Plants.

[41] Hubbard K.E., Siegel R.S., Valerio G., Brandt B., Schroeder J.I. (2011). Abscisic acid and CO_2_ signalling via calcium sensitivity priming in guard cells, new CDPK mutant phenotypes and a method for improved resolution of stomatal stimulus–response analyses. Ann. Bot..

[42] Yang H.M., Zhang J.H., Zhang X.Y. (2005). Regulation Mechanisms of Stomatal Oscillation. J. Integr. Plant Biol..

[43] Gröchenig K. (2001). Foundations of Time-Frequency Analysis.

[44] Kammeyer K.D., Kroschel K. (2012). Digitale Signalverarbeitung.

[45] Farge M. (1992). Wavelet Transforms and their Applications to Turbulence. Ann. Rev. Fluid Mech..

[46] Stockwell R.G. (2007). A basis for efficient representation of the S-transform. Digit. Signal Process..

[47] Huang N.E., Wu Z. (2008). A Review in Hilbert-Huang Transform: Method and its Applications to Geophysical Studies. Rev. Geophys..

[48] Jones H.G. (2007). Monitoring plant and soil water status: Established and novel methods revisited and their relevance to studies of drought tolerance. J. Exp. Bot..

[49] Brogardh T., Johnsson A., Klockare R. (1974). Oscillatory Transpiration and Water Uptake of Avena Plants V. Influences of the Water Potential of the Root Medium. Physiol. Plant..

[50] Vialet-Chabrand S., Matthews J.S.A., McAusland L., Blatt M.R., Griffiths H., Lawson T. (2017). Temporal Dynamics of Stomatal Behavior: Modeling and Implications for Photosynthesis and Water Use. Plant Physiol..

[51] Huang N.E., Wu M.L., Long S.R., Shen S.S., Qu W., Gloersen P., Fan K.L. (2016). A confidence limit for the empirical mode decomposition and Hilbert spectral analysis. Proc. R. Soc. Lond. A.

[52] Prytz G., Futsaether C.M., Johnsson A. (2003). Thermography studies of the spatial and temporal variability in stomatal conductance of Avena leaves during stable and oscillatory transpiration. New Phytol..

[53] Clawson K.L., Blad B.L. (1982). Infrared Thermometry for Scheduling irrigation of Corn. Agron. J..

[54] Schulze E.D. (1986). Carbon Dioxide and Water Vapor Exchangein Response to Drought in the Atmosphere and in the Soil. Ann. Rev. Plant Physiol..

[55] Laleh B., Thomas R.S., Maciej Z., Francesca S., William H., Thomas E.C., Thomas W.R. (2017). Assessing water-related plant traits to explain slow-wilting in soybean PI 471938. J. Crop Improv..

[56] Cowan I.R. (1972). Oscillations in Stomatal Conductance and Plant Functioning Associated with Stomatal Conductance: Observations and a Model. Planta.

[57] Pieruschka R., Huber G., Berry J.A. (2010). Control of transpiration by radiation. Proc. Natl. Acad. Sci. USA.

[58] Curtis O.F. (1936). Leaf Temperatures and the Cooling of Leaves by Radiation. Plant Physiol..

[59] Breidenbach R.W., Saxton M.J., Hansen L.D., Criddle R.S. (1997). Heat Generation and Dissipation in Plants: Can the Alternative Oxidative Phosphorylation Pathway Serve a Thermoregulatory Role in Plant Tissues Other Than Specialized Organs?. Plant Physiol..

